# Delay Differential Equation Approach to Oligomerization Reveals the Delay Dynamics Underlying Oscillations in Mitochondrial Fission

**DOI:** 10.1007/s11538-026-01743-y

**Published:** 2026-08-22

**Authors:** Kitrick Fynaardt, Anna K. Leinheiser, Colleen C. Mitchell, Chad E. Grueter

**Affiliations:** 1 Department of Mathematics, University of Iowa, 2 West Washington Street, Iowa City, IA 52242 USA; 2 Department of Internal Medicine, Division of Cardiovascular Medicine, Francois M. Abboud Cardiovascular Research Center, Fraternal Order of Eagles Diabetes Research Center, University of Iowa, 169 Newton Road, Iowa City, IA 52242 USA

**Keywords:** Mitochondrial dynamics, Mitochondrial fission, Delay-differential equation of threshold type, DDE, Delay-differential equation, Homogenization, Hopf bifurcation, 92C37, 34K60

## Abstract

Disruptions in the balance of mitochondrial fission and fusion are implicated in a host of diseases including cardiovascular, metabolic, and neurodegenerative, as well as cancer. Leinheiser et al. proposed a mechanistic model for mitochondrial fission which relies on the oligomerization of Dynamin-related protein 1 (Drp1). In this work, we propose an alternative state-dependent delay-differential equation (sdDDE) framework for mitochondrial fission, which reveals that the intrinsic delay dynamics in Drp1 oligomerization can drive oscillations in the rate of mitochondrial fission. To develop this sdDDE model, we generate a simplified model which disallows oligomer disassembly on the mitochondrial membrane. Following homogenization, the simplified model approaches a steady state dominated by oligomers too small to reach the threshold for fission. Therefore, the fission rate approaches zero when initial conditions reside within the basin of attraction of this fission-free equilibrium. We therefore reincorporate oligomer disassembly on the mitochondrial membrane. However, the attracting, fission-free equilibrium persists. To eliminate this fission-free equilibrium, we incorporate an atomization term into the oligomerization mechanism, highlighting the importance of oligomer disassembly in sustaining mitochondrial fission. Using homogenization techniques, we derive an advection PDE with nonlocal interactions and obtain a reduced sdDDE system governing oligomer partial moments. Analysis of this reduced system reveals an analogous Hopf bifurcation to the Leinheiser et al. fission model, demonstrating that intrinsic delays in Drp1 oligomerization are sufficient to generate oscillatory mitochondrial fission dynamics.

## Introduction

Mitochondria are organelles that are predominately known for their role in ATP production which is the main energy currency of cells. To maintain homeostasis, mitochondria undergo three main processes - mitophagy, fusion, and fission. This work focuses on a mathematical model of mitochondrial fission which is the process of a single mitochondrion splitting into two daughter mitochondria. In response to cellular stress, mitochondria rapidly fission, creating a large number of small mitochondria. This hyperfission state has been linked to neurodegenerative, cardiovascular, metabolic disease, and cancer (Youle and Bliek 2012; Ponce et al. 2020). For these reasons, understanding the process and regulation of mitochondrial fission is critical.

Leinheiser et al. proposes a model of mitochondrial fission focused on the oligomerization of dynamin related protein 1 (Drp1) (Leinheiser et al. 2024). In their model, Drp1-dependent fission begins when Drp1 binds to mitochondrial fission factor (Mff) on the outer mitochondrial membrane. These Drp1-Mff complexes bind one complex at a time to form oligomers. When an oligomer becomes long enough to wrap around the mitochondrion, it can constrict and facilitate a fission event. The Leinheiser et al. model is a high dimensional system of ordinary differential equations (ODEs) which tracks the concentration of each oligomer size.

**Fig. 1:**
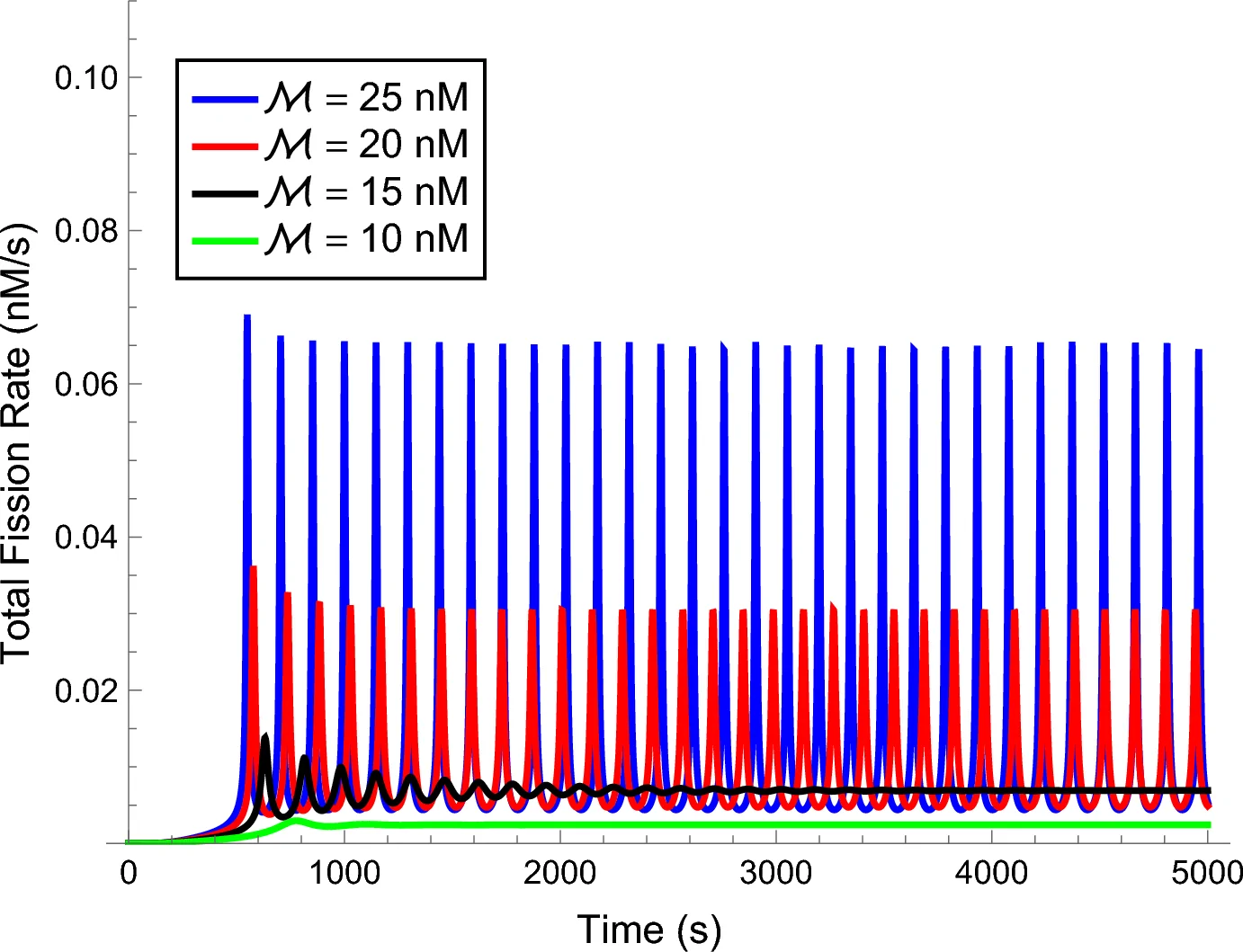
Numerical simulations of the total fission rate of mitochondria predicted by the Leinheiser et al model (Leinheiser et al. 2024). These simulations were run with the parameters $k_+ = 5$, $k_- = 0.1$, ℓ=25, a=5, $k_1 = 1$, $k_{-1} = 0.02$, $\mathcal {T} = 20$, $\mathcal {M} = 15$, γ=0.01, and N=30. The total rate of fission transitions from a stable steady state to a stable oscillation as the pool of available fission proteins passes the Hopf bifurcation

As the total concentration of Mff varies, the total fission rate transitions from damped oscillations that reach an attracting steady state to a solution with stable oscillations, characteristic of a Hopf bifurcation (Fig. 1). Leinheiser et al. go on to confirm this Hopf bifurcation by numerical calculation of the eigenvalues. However, the underlying mechanism which causes these oscillations is still unclear.

In the Leinheiser et al. model, oligomers are built one building block at a time as in a Becker-Döring oligomerization model (Edelstein-Keshet and Ermentrout 1998; Slemrod 2000; Wattis 2006), and it imposes a maximum oligomer size of *N*. The concentrations of Drp1 in the cytosol and Mff on the mitochondrial membrane are denoted *T* and *M* respectively, with initial concentrations $\mathcal {T}$ and $\mathcal {M}$ respectively. They associate at rate $k_1$ to form the building blocks of oligomerization. These are viewed as oligomers of size 1 and therefore denoted $C_1$. An oligomer of size 1 can also dissociate at rate $k_{-1}$. The concentrations of oligomers of size $1\le i\le N$ are denoted as $C_i$. Oligomers of size 1 can associate with an oligomer of size *i* at rate $k_+$ to create an oligomer of size i+1. Any oligomer of size *i* can dissociate at rate $k_-$ into a size 1 oligomer and a size i-1 oligomer. The rate of mitochondrial fission is defined as a piecewise function, *f*(*i*), that depends on oligomer size. Any oligomer of size less than ℓ fissions at rate 0 while oligomers of size greater than or equal to ℓ fission at a constant rate *a*. This setup imposes a minimum oligomer size before fission can occur, consistent with the intuition that oligomers must be long enough to encircle the mitochondrion.

Drp1 is released from the endoplasmic reticulum following acute cellular stress (Osterlund et al. 2023). To simulate this sudden influx of oligomer building material, we choose the initial conditions $$T(0) = \mathcal {T}$$, $$M(0) = \mathcal {M}$$, and $$ C_i(0) = 0$$, $$1 \le i \le N$$. That is, there are no oligomers of any size on the mitochondrial surface, and there is an initial influx of Drp1 that can associate with the Mff present on the mitochondrial surface. This choice of initial conditions is also consistent with the simulations in Leinheiser et al. The full model is

1a $$\begin{aligned} \dot{T}&= \gamma \left( k_{-1} C_1 - k_1 T M + \sum _{i = 1}^N i f(i) C_i \right) , \end{aligned}$$

1b $$\begin{aligned} \dot{M}&= k_{-1} C_1 - k_1 T M + \sum _{i = 1}^N i f(i) C_i, \end{aligned}$$

1c $$\begin{aligned} \dot{C}_1&= k_1 T M - k_{-1} C_1 + k_- \left( 2 C_2 + \sum _{i = 3}^N C_i \right) - k_+ \left( 2 C_1^2 + \sum _{i = 2}^{N - 1} C_i C_1 \right) , \end{aligned}$$

1d $$\begin{aligned} \dot{C}_i&= k_+ C_{i - 1}C_1 + k_- C_{i + 1} - k_- C_i - k_+ C_i C_1 - f(i) C_i, \quad 2 \le i < N, \end{aligned}$$

1e $$\begin{aligned} \dot{C}_N&= k_+ C_{N-1} C_1 - k_- C_N - f(i) C_N, \end{aligned}$$

1f $$\begin{aligned} f(i)&= {\left\{ \begin{array}{ll} 0, \quad 1 \le i \le \ell , \\ a, \quad \ell < i \le N, \end{array}\right. } \end{aligned}$$

1g $$\begin{aligned} T(0)&= \mathcal {T}, \quad M(0) = \mathcal {M}, \quad C_i(0) = 0 \quad 1 \le i \le N. \end{aligned}$$

In this work, we propose an alternative state-dependent delay-differential equation (sdDDE) model of threshold type which reveals the inherent delay dynamics associated with mitochondrial fission. Our goal is to introduce a model which retains each of the qualitative behaviors of the original model while giving insight into the underlying mechanisms. In the development of this sdDDE model, we generate a simplified model which disallows oligomer disassembly on the mitochondrial membrane. Following homogenization, the simplified model in Sect. 2 has two steady states, one of which is dominated by medium-sized oligomers, and as a consequence, fission never occurs. We prove our initial conditions lie in the basin of attraction of this fission-free equilibrium. We confirm the existence of a stalled wave similar to the Leinheiser et al. model. Since a model without fission is not biologically accurate, we reincorporate oligomer disassembly in Sect. 3 with a bidirectional model. However, the stable fission-free equilibrium seen in the previous section persists after homogenization. To eliminate this fission-free equilibrium, we replace dissociation with an atomization term in Sect. 4. As a result, we eliminate the fission-free equilibrium and show the homogenized atomization model is equivalent to a sdDDE. This model has an analogous Hopf bifurcation to the original Drp1-dependent fission model.

## A Simplified Model

**Fig. 2:**
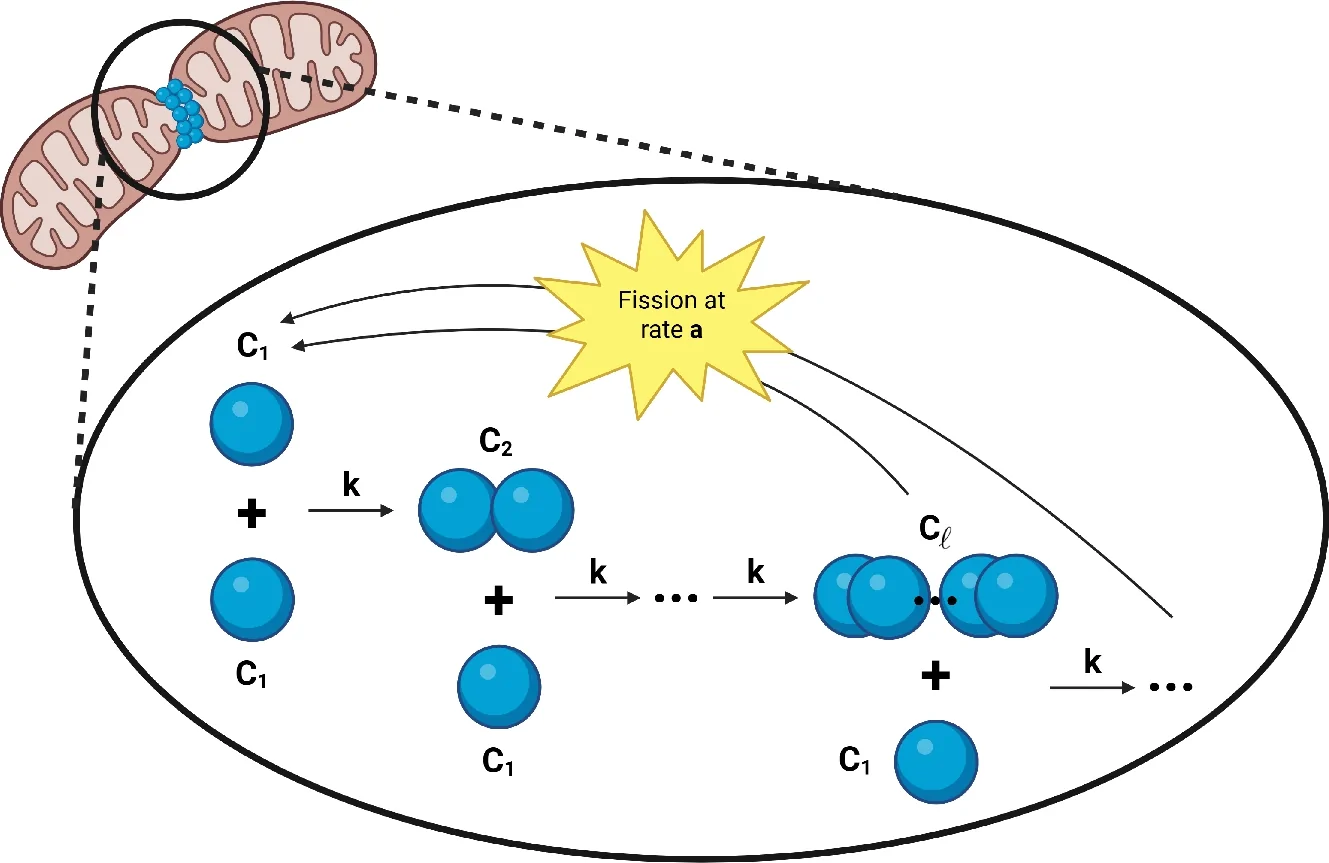
The reaction schematic for a simplified model of mitochondrial fission. The initial condition $C_1(0) = Q$ represents an initial cohort of oligomers of size 1. These associate at rate *k* into oligomers of size 2. Oligomers of size *i* combine with oligomers of size 1 at rate *k* to create oligomers of size i+1. Oligomers of size ℓ or larger can initiate fission at rate *a*

We begin by making three simplifications to the Leinheiser et al. model (Leinheiser et al. 2024). First, we assume there is no maximum size for oligomers. This approximation is reasonable because concentrations of oligomers larger than size ℓ are small enough that they do not substantially contribute to the sums in (1c) (Leinheiser et al. 2024). Second, we omit the dissociation rate of oligomers. This choice is motivated by the intuition that the growth of oligomers on the membrane is more important than their disassembly. This change will be revisited in the next section. Third, we omit the dynamics characterizing Drp1 and Mff interactions. These variables (*M* and *T*) are eliminated because in the Leinheiser et al. model, the concentration of cytosolic Drp1 varies minimally over time, and the limiting factor is the concentration of Mff. As a result, the oligomerization process begins with the pool of Drp1-Mff building blocks ($C_1$) as opposed to the initial interaction of Drp1 and Mff (Fig. 2). The notation for this system is analogous to the previous with the exceptions that the association rate of oligomers with building blocks is simply *k*, the initial concentration of building blocks is *Q*, and f(ℓ)=a to shift the discontinuity from ℓ+1 to ℓ. This gives us the following system of equations:

2a $$\begin{aligned} \dot{C}_{1 }&= a\sum _{i = \ell }^\infty i C_i - k\left( C_{1}^{2} + \sum ^{\infty }_{i=1}C_{i}C_{1} \right) , \end{aligned}$$

2b $$\begin{aligned} \dot{C}_{i}&= kC_{1} C_{i-1} - kC_1C_i - f(i) C_i, \quad i \ge 2, \end{aligned}$$

2c $$\begin{aligned} f(i)&= {\left\{ \begin{array}{ll} 0, & i < \ell , \\ a, & i \ge \ell , \end{array}\right. } \end{aligned}$$

2d $$\begin{aligned} C_1(0)&= Q, \quad C_i(0) = 0, \quad i > 1. \end{aligned}$$

In this system, the total concentration of building blocks, $\sum _{i=1}^\infty i C_i$, is conserved. Therefore,

$$\begin{aligned} \sum _{i=1}^\infty i C_i = C_1(0) = Q. \end{aligned}$$

### Homogenizing the Simplified Model

System (2) becomes unwieldy as ℓ becomes large because the dimension of the system scales with ℓ. In addition, the steady state solutions include polynomials of degree ℓ which are difficult to estimate numerically (Leinheiser 2023). We can approximate the behavior of this model by converting oligomer size into a continuous variable of a multivariate function. An example of such an approximation can be found in Batkai et al. (2015). We introduce $\Theta (i,t) = C_i(t)$ for i≥1. Therefore, Equation (2b) is now the dynamics of Θ(i,t), and Equation (2a) is the left boundary condition. For ease of notation, we will say $C_1(t) = \Theta (1,t) = C$. As a result, the homogenized simplified model is

3a $$\begin{aligned} \dot{C}&= a \int _\ell ^\infty u \Theta (u,t) du - k C^2 - k C \int _1^\infty \Theta (u,t) du, \end{aligned}$$

3b $$\begin{aligned} \frac{\partial \Theta }{\partial t}&= - k C \frac{\partial \Theta }{\partial s} - f(s) \Theta (s,t), \quad s > 1, \end{aligned}$$

3c $$\begin{aligned} \Theta (1,t)&= C(t). \end{aligned}$$

The conserved quantity is similarly homogenized:

4 $$\begin{aligned} C + \int _1^\infty u \Theta (u,t) du = Q. \end{aligned}$$

Note that *Q* is finite which implies $\int _1^\infty u \Theta (u,t) du$ and $\int _1^\infty \Theta (u,t)du$ are bounded. This is biologically sound.

System (3) has two steady states, one is a fission-free equilibrium:

5a $$\begin{aligned} C&= 0, \end{aligned}$$

5b $$\begin{aligned} \Theta (s)&= h(s),&s < \ell , \end{aligned}$$

5c $$\begin{aligned} \Theta (s)&= 0,&s \ge \ell . \end{aligned}$$

where $h: \mathbb {R} \rightarrow \mathbb {R}$ is any function such that h(1)=0 and $\int _1^{\ell } s \, h(s) ds=Q$. This is a “fission-free" equilibrium since the concentration of building blocks, *C*, is zero, and the concentration of oligomers large enough to facilitate fission, $s \ge \ell$, is also zero. In other words, all building blocks are caught up in middle sized oligomers and the building process stalls.

The other steady state is

6a $$\begin{aligned} C&= \frac{- \frac{1}{2}(\ell ^2 + 1) + \sqrt{\frac{1}{4}(\ell ^2 + 1)^2 + 4 \frac{kQ}{a}}}{2\frac{k}{a}}, \end{aligned}$$

6b $$\begin{aligned} \Theta (s)&= C,&s < \ell , \end{aligned}$$

6c $$\begin{aligned} \Theta (s)&= C e^{\frac{-a}{k C}(s - \ell )},&s \ge \ell . \end{aligned}$$

Note that the total amount of fission occurring at this steady state, called the total fission rate, can be calculated with $\int _\ell ^\infty a \Theta (u)du$ (Leinheiser et al. 2024). The total fission rate of the above equilibrium is $\int _\ell ^\infty a \Theta (u)du = k C^2$.

#### Discontinuities in the Fission Function and Initial Conditions

In Equation (3b), we choose *f*(*s*) to be the same piecewise function as in System (2):

7 $$\begin{aligned} f(s) = {\left\{ \begin{array}{ll} 0, \quad s < \ell , \\ a, \quad s \ge \ell . \end{array}\right. } \end{aligned}$$

This choice of *f*(*s*) introduces a discontinuity to Equation (3b). Therefore, we solve the PDE for $s \in [1, \ell ]$ and use $\Theta (\ell ,t)$ as the boundary condition for the PDE from $(\ell ,\infty )$.

Furthermore, we wish to impose initial conditions analogous to System (2). Recall these initial conditions are chosen to reflect a sudden release of Drp1 from the endoplasmic reticulum, causing an initial impulse of oligomer building material. With the concentrations of Drp1 and Mff removed from the simplified model, we can simulate the release from the endoplasmic reticulum as an initial count of oligomers of size one, with all other sizes initialized to zero.

8a $$\begin{aligned} \Theta (1,0)&= C(0) = Q, \end{aligned}$$

8b $$\begin{aligned} \Theta (s,0)&= 0, \quad s > 1. \end{aligned}$$

This choice introduces another discontinuity which begins at (1, 0) and propagates through the solution. Therefore, any solution with these initial conditions would solve System (3) in the weak sense. Note, the PDE holds on either side of the discontinuity caused by the initial conditions.

Let $s^*(t)$ be the size where the discontinuity propagated from the initial conditions appears. We take Θ(s,t) to be left continuous and therefore $\Theta (s^*,t) = Q$ when $s^* < \ell$. Furthermore Θ(s,t)=0 for $s > s^*$. We use the Leibniz rule, Equation (3a), and Equation (3b) to differentiate Equation (4):

9a $$\begin{aligned} Q&= C + \int _1^{\infty } u \Theta (u,t)du = C + \int _1^{s^*} u \Theta (u,t)du, \end{aligned}$$

9b $$\begin{aligned} 0&= \dot{C} + \int _1^{s^*} u \frac{\partial \Theta }{\partial t} du + s^* \Theta (s^*, t )\frac{ds^*}{dt}, \end{aligned}$$

9c $$\begin{aligned} 0&= - k C^2 - k C \int _1^{s^*} \Theta (u,t) du - k C \int _1^{s^*} u \frac{ \partial \Theta }{\partial u} du + s^* Q \frac{ds^*}{dt}, \end{aligned}$$

9d $$\begin{aligned} 0&= - kC^2 -kC \int _1^{s^*} \Theta (u,t) du \end{aligned}$$

9e $$\begin{aligned}&\quad - k C \left( s^* \Theta (s^*, t) - C - \int _1^{s^*} \Theta (u,t) du \right) + s^* Q \frac{ds^*}{dt}, \quad \text {(Int. by parts)}, \end{aligned}$$

9f $$\begin{aligned} 0&= -k C s^* Q + s^* Q \frac{d s^*}{dt}, \end{aligned}$$

9g $$\begin{aligned} \frac{ds^*}{dt}&= k C. \end{aligned}$$

We further define $t^*$ using integration:

10a $$\begin{aligned} s^*(t^*) - s^*(0)&= \ell - 1 \end{aligned}$$

10b $$\begin{aligned}&= \int _{0}^{t^*} \frac{ds^*}{d\xi } d\xi = \int _{0}^{t^*} k C(\xi ) d \xi \end{aligned}$$

Intuitively, $t^*$ is the time where the discontinuity created by the initial conditions crosses the discontinuity at size ℓ. We note there is no guarantee such a $t^*$ exists.

Due to the nonlocal interactions in the PDE boundary condition and velocity of the traveling wave, we define

11 $$\begin{aligned} n(t) = \int _1^\infty \Theta (u,t) du. \end{aligned}$$

For $s^*(t) < \ell$, the impulse created by the initial conditions has only traveled to $s^* < \ell$, so

12 $$\begin{aligned} n(t) = \int _1^{s^*} \Theta (u,t)du. \end{aligned}$$

Then we can use the Leibniz rule to write,

13 $$\begin{aligned} \dot{n}(t)&= \int _1^{s^*} \frac{\partial \Theta (u,t)}{\partial t} du + \frac{ds^*}{dt} \Theta (s^*,t) \nonumber \\&= - kC \int _1^{s^*} \frac{\partial \Theta (u,t)}{\partial s}du + k C Q \nonumber \\&= k C^2. \end{aligned}$$

### A Stalled Wave

For $s^*(t) < \ell$, System (3), with Equation (13), and initial conditions (8) create a new ODE system. Note for $s^*(t) < \ell$, the first integral term in Equation (3a) is 0. Then

14a $$\begin{aligned} \dot{C}&= -k C^2 - k C n, \end{aligned}$$

14b $$\begin{aligned} \dot{n}&= k C^2, \end{aligned}$$

14c $$\begin{aligned} C(0)&= Q, \quad n(0) = 0. \end{aligned}$$

**Fig. 3:**
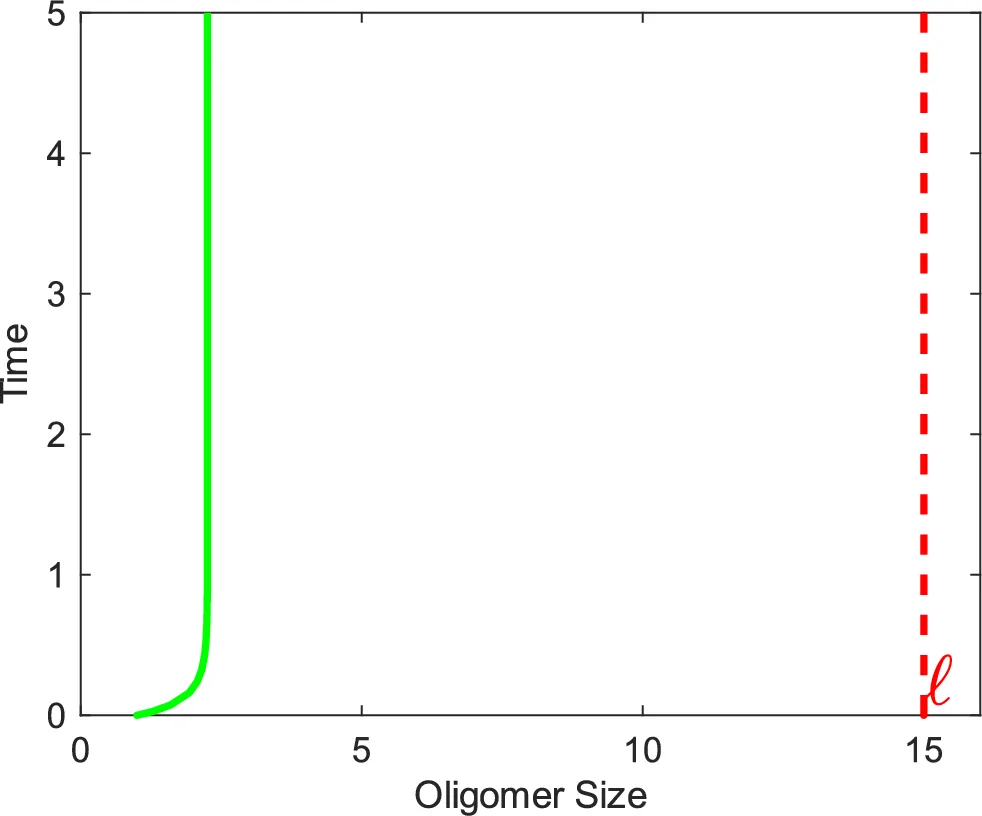
A graph of $s^*(t)$ in the homogenized simplified model with parameters ℓ=15, b=0.02, k=1, and Q=12. Note $s^*$ does not travel far in the size direction and never reaches the red dotted line of ℓ=15

This system can be solved for *C* and plugged into $\int _0^{t^*} k C(\xi ) d\xi = \ell - 1$ to solve for $t^*$. However, there is no guarantee a solution $t^*$ exists. If there is no solution, the wave defined by Equation (3b) never reaches size ℓ, and the method of characteristics produces a stalled wave (Fig. 3). With this stalled wave, the impulse created by the initial conditions never reaches size ℓ, implying we approach the fission-free steady state. The following theorem proves there is no solution for $t^*$ when ℓ>3.

#### Theorem 1

If k>0, Q>0, and *C*(*t*) is a solution to System (14), then

$$\begin{aligned} \int _0^\infty k C(\xi ) d\xi < 2. \end{aligned}$$

#### Proof

Let *C*(*t*) and *n*(*t*) be solutions to System (14). Note that if C(t)=0 for any *t*, $\dot{C}(t) = 0$, and therefore *C*(*t*) cannot cross the *t*-axis. Since C(0)=Q>0, C(t)>0 for t≥0. Since $\dot{n} = k C(t)^2$, *n*(*t*) is increasing. We seek to define new ODEs with closed-form solutions that are guaranteed to be larger than *C*(*t*) for all *t*. Note that k>0 implies $\dot{C} = -k C^2 - k C n \le -k C^2$ for t≥0. We can use this fact to define dynamics on a new ODE that has a derivative always larger than *C*(*t*) (but still negative).

Define *A*(*t*) to be the solution to

15a $$\begin{aligned} \dot{A}&= -k A^2, \end{aligned}$$

15b $$\begin{aligned} A(0)&= Q. \end{aligned}$$

Since *C*(*t*) and *A*(*t*) have the same initial condition and $\dot{C}(t) \le \dot{A}(t)$ for t≥0, we know C(t)≤A(t) for t≥0. The solution is $A(t) = \frac{Q}{k Q t + 1}$. Thus,

16 $$\begin{aligned} C(t) \le \frac{Q}{k Q t + 1}, \quad t \ge 0. \end{aligned}$$

This *A*(*t*) is one of the closed-form solutions we sought. However, the integral of *A*(*t*) is unbounded. Therefore, we define yet another ODE with a closed-form solution that is guaranteed to be larger than *C*(*t*) for *t* greater than some non-zero time. Fix time α>0. Since *n*(*t*) is increasing, we know n(α)>0 for any α>0. This allows us to define *B*(*t*) to be the solution to

17a $$\begin{aligned} \dot{B}&= -k B n(\alpha ), \end{aligned}$$

17b $$\begin{aligned} B(\alpha )&= \frac{Q}{k Q\alpha + 1}. \end{aligned}$$

Since *n*(*t*) is increasing, $\dot{C}(t) \le \dot{B}(t)$ for $t \ge \alpha$. Then because $B(\alpha ) = A(\alpha ) \ge C(\alpha )$, we know C(t)≤B(t) for $t \ge \alpha$. The solution is $B(t) = Q e^{k n(\alpha )(\alpha - t)}/(k Q \alpha + 1)$. Thus,

18 $$\begin{aligned} C(t) \le \frac{Q e^{k n(\alpha )(\alpha - t)}}{k Q \alpha + 1}, \quad t \ge \alpha . \end{aligned}$$

Thus, we have two equations which we can integrate to bound the integral of *kC*(*t*). The first, *A*(*t*), is a bound on *C*(*t*) which we cannot use for $t \rightarrow \infty$ because its integral is unbounded. So, we use bound *A*(*t*) until time α. At t=α we can use the fact that n(α)>0 to define bound *B*(*t*) which we use for $t \ge \alpha$.

By Equations (16) and (18), and the continuity of the integral, we get

19 $$\begin{aligned} \int _0^\infty k C(\xi ) d\xi&\le \int _0^\alpha \frac{k Q}{k Q \xi + 1} d\xi + \int _\alpha ^\infty \frac{k Q e^{k n(\alpha )(\alpha - \xi )}}{k Q \alpha + 1} d\xi \nonumber \\&= \ln (k Q \alpha + 1) + \frac{Q}{(k Q \alpha + 1) n(\alpha )}. \end{aligned}$$

Since n(α) appears in the denominator of this bound, we need an underestimate of n(α). We use a similar strategy, defining a new ODE with a closed-form solution whose solution will always be smaller than *n*(*t*). Then we can use this new solution evaluated at α in the bound of the integral of *kC*(*t*).

Define *F*(*t*) and *g*(*t*) to be the solutions to the ODE system

20a $$\begin{aligned} \dot{F}&= -k Q F - k F g, \end{aligned}$$

20b $$\begin{aligned} \dot{g}&= k F^2, \end{aligned}$$

20c $$\begin{aligned} F(0)&= Q, \quad g(0) = 0. \end{aligned}$$

Recall, Q≥C(t) for t≥0. So $\dot{F}(t) \le \dot{C}(t)$ and thus F(t)≤C(t) for t≥0. So, g(t)≤n(t) for t≥0. The solutions for *F*(*t*) and *g*(*t*) are

21a $$\begin{aligned} F(t)&= \sqrt{2} Q \sqrt{1 - \tanh ^2(\sqrt{2} k Q t + \text {arccosh}(-\sqrt{2}))}, \end{aligned}$$

21b $$\begin{aligned} g(t)&= -Q + \sqrt{2} Q \tanh (\sqrt{2} k Q t + \text {arccosh}(- \sqrt{2})). \end{aligned}$$

Define $\alpha ^* = k Q \alpha$. By (19) and (21b):

$$\begin{aligned}&\int _0^\infty k C(\xi ) d\xi \le \ln (k Q \alpha + 1) + \frac{Q}{(k Q \alpha + 1) n(\alpha )} \\&\le \ln (k Q \alpha + 1) + \frac{Q}{(k Q \alpha + 1) (-Q + \sqrt{2} Q \tanh (\sqrt{2} k Q \alpha + \text {arccosh}(- \sqrt{2})))} \\&= \ln (\alpha ^* + 1) + \frac{1}{(\alpha ^* + 1) (-1 + \sqrt{2} \tanh (\sqrt{2} \alpha ^* + \text {arccosh}(- \sqrt{2})))}. \end{aligned}$$

Parameters *k* and *Q* affect the value of α where the bound is minimized, but they do not effect the minimum value. Recall the bound holds for any α>0 and therefore any $\alpha ^* > 0$. One can calculate that when $\alpha ^* = 1.65$ the bound is equal to 1.89571<2. Thus,

$$\begin{aligned} \int _0^\infty k C(\xi ) d\xi&< 2. \end{aligned}$$

□

By this theorem, $\int _0^{t^*} k C(\xi ) d\xi = \ell -1$ has no solution for $t^*$ when ℓ>3. The theorem makes use of the chosen initial conditions for the simplified model. These initial conditions were chosen to be analogous to the Leinheiser et al. model, but we note that different initial conditions may escape the basin of attraction of the fission-free, stalled-wave equilibrium. Such initial conditions could be investigated in future work.

**Fig. 4:**
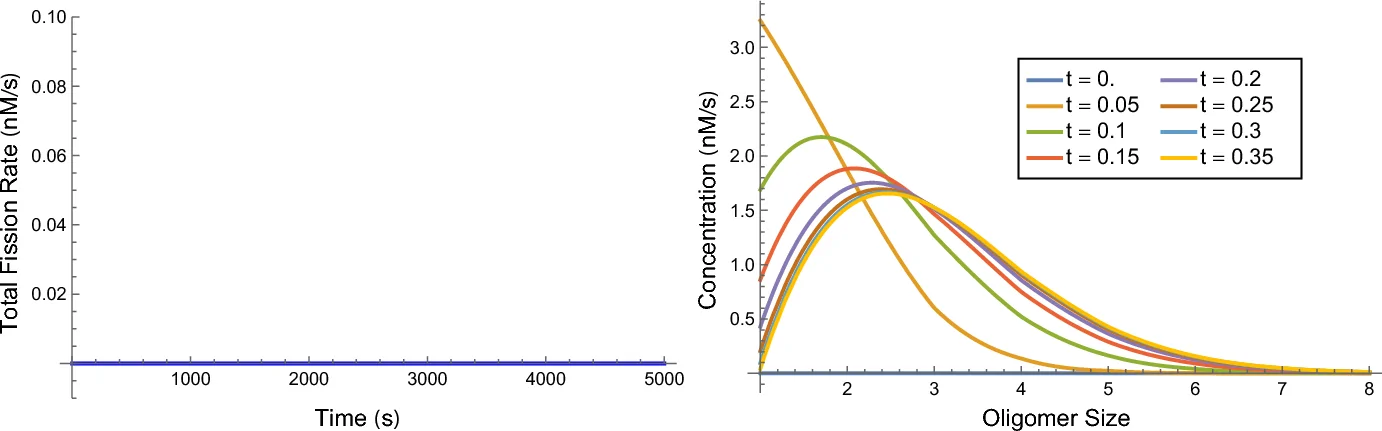
A numerical simulation of the Leinheiser et al. model with parameters $k_+ = 5$, $k_- = 0$, ℓ=25, a=5, $k_1 = 1$, $k_{-1} = 0.02$, $\mathcal {T} = 20$, $\mathcal {M} = 15$, γ=0.01, and N=30. The left figure shows there is no fission with these parameters. The right figure shows snapshots of the size distribution interpolated between integer sizes at various time steps. The concentrations of sizes beyond s=8 are omitted, as they are all 0. The middle sizes of oligomers stall as early as t=0.35, which is consistent with the findings from the simplified model

Based on the above theorem, we conjectured that a stalled wave exists in the Leinheiser et al. model when $k_- = 0$. Indeed, numerical simulations with $k_-=0$ confirm that without dissociation of some kind, the Leinheiser et al. model also exhibits a stalled-wave equilibrium where fission does not occur (Fig. 4).

## The Bidirectional Model

**Fig. 5:**
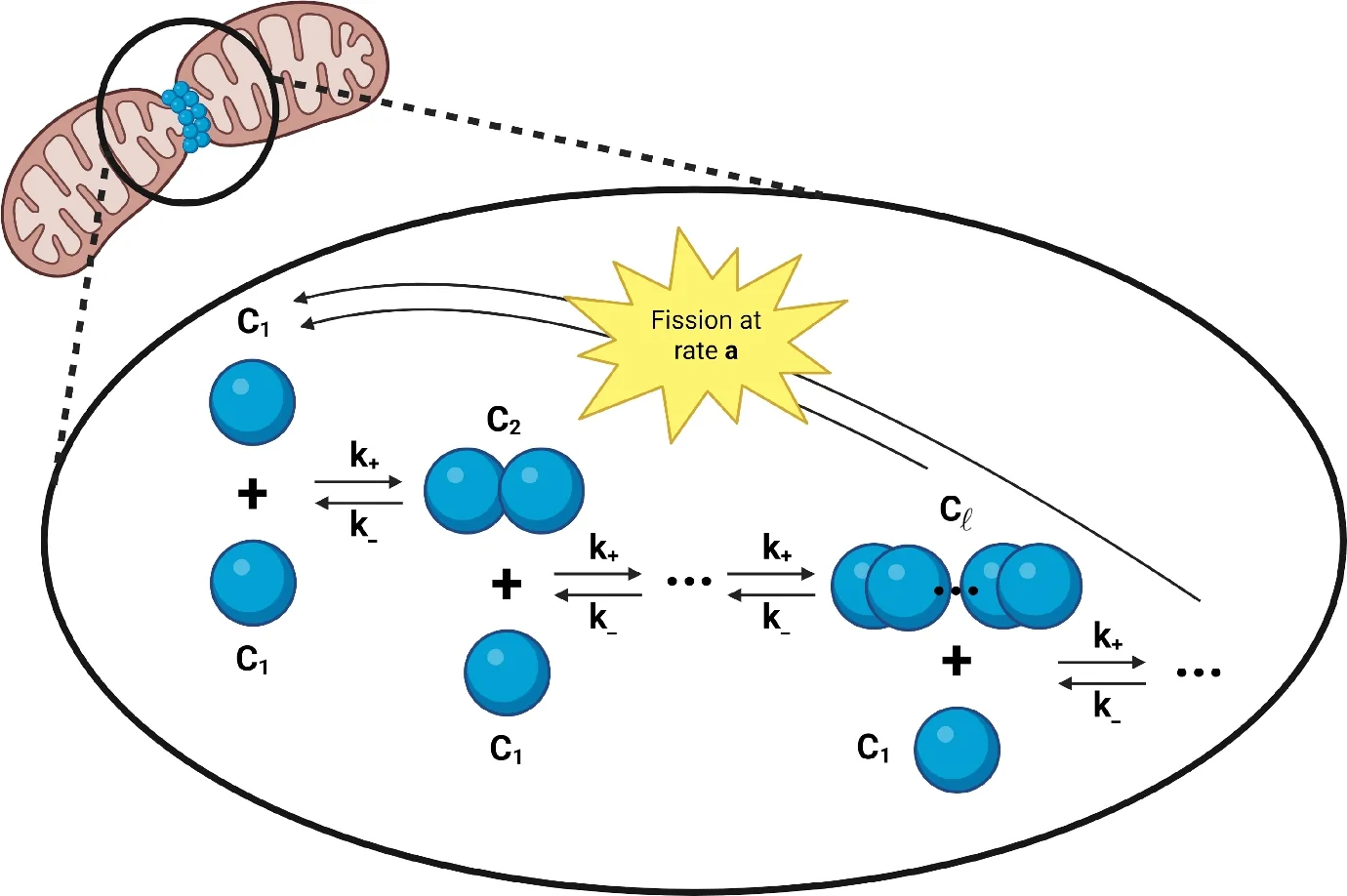
The reaction schematic for a bidirectional model of mitochondrial fission. Oligomers of size 1 ($C_1$) combine into larger oligomers at the rate $k_+$ and separate at the rate $k_-$. Once oligomers reach size ℓ or larger, they can initiate a fission event at rate *a*

To remedy the stalled wave phenomenon discussed in Sect. 2.2, we reintroduce the dissociation rate $k_-$ from the Leinheiser et al. model (Fig. 5). This produces the bidirectional model:

22a $$\begin{aligned} \dot{C}_1&= a \sum _{i = \ell }^\infty i C_i - k_+ \left( C_1^2 + \sum _{i = 1}^{\infty } C_i C_1 \right) + k_- \left( C_2 + \sum _{i = 2}^{\infty } C_i \right) , \end{aligned}$$

22b $$\begin{aligned} \dot{C}_i&= k_+ C_1 C_{i - 1} - k_+ C_1 C_i - k_- C_i + k_- C_{i + 1} - f(i) C_i, \quad i \ge 2, \end{aligned}$$

22c $$\begin{aligned} f(i)&= {\left\{ \begin{array}{ll} 0, & i < \ell , \\ a, & i \ge \ell , \end{array}\right. } \end{aligned}$$

22d $$\begin{aligned} C_1(0)&= Q, \quad C_i(0) = 0, \quad i>1. \end{aligned}$$

The total number of size 1 oligomers is still conserved:

$$\begin{aligned} \sum _{i = 1}^\infty i C_i = C_1(0) = Q. \end{aligned}$$

### Homogenizing the Bidirectional Model

Similar to the simplified model (System (2)), the bidirectional model (System (22)) becomes unwieldy as ℓ becomes large. We homogenize following the same procedure described in Sect. 2.1. The resulting homogenized bidirectional model is

23a $$\begin{aligned} \dot{C}&= a \int _\ell ^\infty u \Theta (u,t)du - (k_+ C - k_-) \left( C + \int _1^\infty \Theta (u,t) du \right) , \end{aligned}$$

23b $$\begin{aligned} \frac{\partial \Theta }{\partial t}&= -(k_+ C - k_-) \frac{\partial \Theta }{\partial s} - f(s) \Theta (s,t), \quad s > 1. \end{aligned}$$

with the conserved quantity

$$\begin{aligned} C + \int _1^\infty u \Theta (u,t) du = Q. \end{aligned}$$

Note that *Q* is finite which implies $\int _1^\infty u \Theta (u,t) du$ and $\int _1^\infty \Theta (u,t)du$ are bounded. This is biologically sound.

We note here a critical step that was taken when homogenizing Equation (22b). We chose to use both a forward and backward difference approximation for the partial derivative:

$$\begin{aligned} C_{i+1} - C_i \approx \frac{\partial \Theta }{\partial s} \approx C_i - C_{i - 1}. \end{aligned}$$

This choice was made because using only forward, backward, or center difference when homogenizing produces a diffusion term that disallows the use of the method of characteristics in later steps.

System (23) has two steady states, one is a fission-free equilibrium:

24a $$\begin{aligned} C&= \frac{k_-}{k_+}, \end{aligned}$$

24b $$\begin{aligned} \Theta (s)&= h(s),&s < \ell , \end{aligned}$$

24c $$\begin{aligned} \Theta (s)&= 0,&s \ge \ell . \end{aligned}$$

where $h: \mathbb {R} \rightarrow \mathbb {R}$ is any function such that $h(1) = \frac{k_-}{k_+}$ and $C + \int _1^{\ell } s \, h(s) ds=Q$. The other steady state is

25a $$\begin{aligned} C&= \frac{\left( \frac{k_-}{a} - \frac{\ell ^2}{2} - \frac{1}{2}\right) + \sqrt{\left( \frac{\ell ^2}{2} + \frac{1}{2} - \frac{k_-}{a}\right) ^2 + 4 \frac{k_+ Q}{a}}}{2 \frac{k_+}{a}}, \end{aligned}$$

25b $$\begin{aligned} \Theta (s)&= C,&s < \ell , \end{aligned}$$

25c $$\begin{aligned} \Theta (s)&= C e^{\frac{-a}{k_+ C - k_-}(s - \ell )},&s \ge \ell . \end{aligned}$$

The total fission rate of this equilibrium is $\int _\ell ^\infty a \Theta (u,t)du = C(k_+ C - k_-)$. We will determine if our initial conditions once again lie in the basin of attraction for the fission-free equilibrium.

#### Discontinuities in the Fission Function and Initial Conditions

We choose the fission function to be Equation (7), and we choose the initial conditions to be Equation (8). Any solution with these initial conditions solves System (23) in the weak sense. We define $s^*$ and $t^*$ analogously to Sect. 2.1.1. Then, for $s^*(t) < \ell$, we define

26 $$\begin{aligned} n(t) = \int _1^\infty \Theta (u,t)du = \int _1^{s^*} \Theta (u,t)du \end{aligned}$$

and find

27 $$\begin{aligned} \dot{n}(t) = (k_+ C - k_-) C. \end{aligned}$$

### A Stalled Wave persists in the Homogenized Bidirectional Model

For $s^*(t) < \ell$, System (23), with Equation (27), and initial conditions (8) create the new ODE system:

28a $$\begin{aligned} \dot{C}&= -(k_+ C - k_-)(C + n), \end{aligned}$$

28b $$\begin{aligned} \dot{n}&= (k_+ C - k_-)C, \end{aligned}$$

28c $$\begin{aligned} C(0)&= Q, \quad n(0) = 0. \end{aligned}$$

This system can be solved for *C* and used in $\int _0^{t^*} k_+ C(\xi ) - k_- d\xi = \ell - 1$ to solve for $t^*$. The following theorem shows there is no solution for reasonable ℓ.

#### Theorem 2

If $k_+ > 0$, $k_- > 0$, $Q \ge \frac{k_-}{k_+}$, and *C*(*t*) is a solution to System (28), then

$$\begin{aligned} \int _0^\infty k_+ C(\xi ) - k_- d\xi \le \ln \left( \frac{Q k_+}{k_-} \right) . \end{aligned}$$

#### Proof

Let *C*(*t*) and *n*(*t*) be solutions to System (28). Note that if $C(t) = \frac{k_-}{k_+}$ for any t, $\dot{C}(t) = 0$, and therefore *C* cannot cross the horizontal line at $\frac{k_-}{k_+}$. Since $C(0) = Q \ge \frac{k_-}{k_+}$, that means $C(t) > \frac{k_-}{k_+}$ for t≥0. Since $\dot{n} = (k_+C - k_-)C$ and $\frac{k_-}{k_+} > 0$, *n*(*t*) is increasing. We seek to define a new ODE with a closed-form solution that is guaranteed to be larger than *C*(*t*) for all *t*. Note that $\dot{C} = -(k_+ C - k_-)(C + n_1) \le -(k_+ C - k_-)C$ for t≥0. We can use this fact to define dynamics on a new ODE that has a derivative always larger than *C*(*t*) (but still negative).

Define *A*(*t*) to be the solution to

29a $$\begin{aligned} \dot{A}&= -(k_+A - k_-)A, \end{aligned}$$

29b $$\begin{aligned} A(0)&= Q. \end{aligned}$$

Since *C*(*t*) and *A*(*t*) have the same initial conditions and $\dot{C}(t) \le \dot{A}(t)$ for t≥0, we know C(t)≤A(t) for t≥0. The solution is:

$$\begin{aligned} A(t) = \frac{k_- Q e^{k_- t}}{k_- + k_+ Q (e^{k_- t} - 1)}. \end{aligned}$$

Thus,

$$\begin{aligned} C(t) \le \frac{k_- Q e^{k_- t}}{k_- + k_+ Q (e^{k_- t} - 1)}, \quad \text {for} \ t \ge 0. \end{aligned}$$

By continuity of the integral,

30a $$\begin{aligned} \int _0^\infty k_+ C(\xi ) - k_- d\xi&\le \int _0^\infty k_+ A(\xi ) - k_- d\xi \end{aligned}$$

30b $$\begin{aligned}&= \lim _{t \rightarrow \infty } \ln {\left( \frac{k_+ Q (e^{ t k_-} - 1) + k_-}{k_-} \right) } - t k_- \end{aligned}$$

30c $$\begin{aligned}&= \ln \left( \frac{Q k_+}{k_-} \right) . \end{aligned}$$

□

At the nominal values from Leinheiser et al. (2024), Q=20, $k_+ = 5$, $k_- = 0.1$, $\ln \left( \frac{Q k_+}{k_-}\right) \approx 6.9$. Thus we prove the wave stalls for $\ell \ge 8$. Numerical evidence finds the wave actually stalls for ℓ as small as 2.

The form of the bound in this theorem gives the impression that small $k_-$ or even $k_- = 0$ may cause the integral defining $t^*$ to grow without bound. However, this theorem is not bidirectional. The bound being larger does not imply the integral will reach that bound, or even grow larger at all. In fact, the $k_- = 0$ case is equivalent to the previous section: the simplified model, which also had a stalled wave. This discourages the idea that small $k_-$ removes the stalled-wave in the bidirectional model. Note that because the wave speed $k_+ C - k_-$ includes a negative term, the above argument needed only one estimate of the solution *C*(*t*), unlike the proof of Theorem (1) which needed two estimates for *C*(*t*) and an estimate for *n*(*t*).

Therefore, the stalled wave persists in the homogenized bidirectional model. Note that in the Leinheiser et al. model, $k_- > 0$ remedies the stalled wave issue. Yet the addition of $k_-$ in the bidirectional model does not have the same effect. We conjecture this inconsistency is from our choice in homogenizing Equation (22b), where a “hybrid" between the backward and forward difference approximations was used to remove diffusion from the resulting homogenization. We further conjecture that a homogenized version of the bidirectional model which includes diffusion may also avoid the stalled wave equilibrium for the given initial conditions. However, with diffusion included in the homogenized bidirectional system, there is no obvious analog to using the method of characteristics to track the discontinuity of the initial conditions. Therefore, we leave it to future work to investigate a homogenized bidirectional model which includes diffusion. Instead, we continue with a change to the model that removes the fission-free equilibrium altogether, and recovers an analogous Hopf bifurcation to the Leinheiser et al. model.

## The Atomization Model

**Fig. 6:**
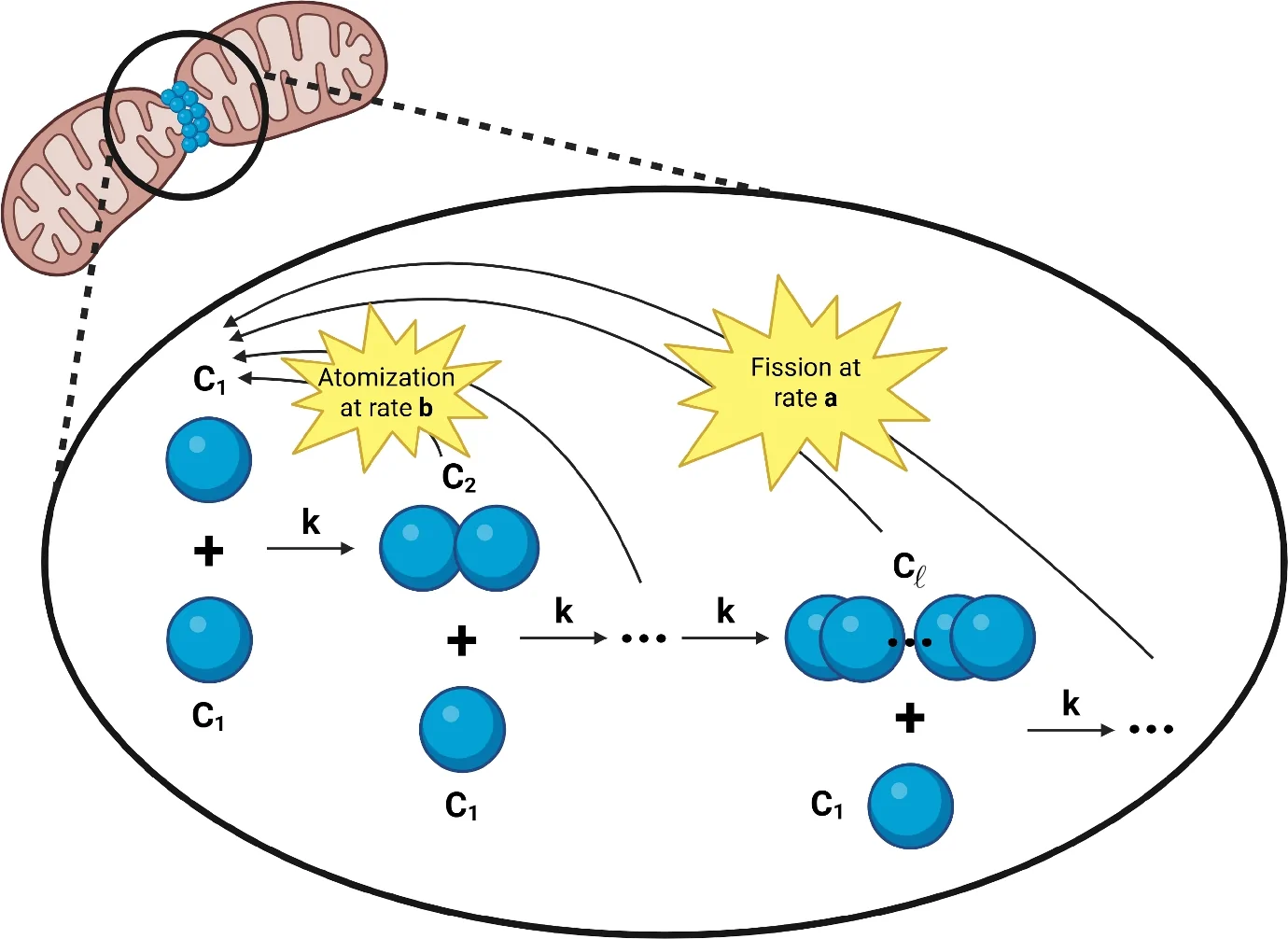
The reaction schematic for the atomization model of mitochondrial fission. Fission complexes combine into oligomers at rate *k* and atomize back to size 1 at rate *b*. Oligomers cause a fission event at rate *a* once they reach size ℓ or larger

In order to guarantee the existence of $t^*$ for any ℓ, we propose the atomization model. The atomization model once again removes $k_-$ and introduces a new parameter *b*, the atomization parameter. The atomization process does not deconstruct an oligomer one building block at a time, but instead releases all building blocks all at once (Fig. 6). This atomization process is separate from a fission event.

31a $$\begin{aligned} \dot{C}_1&= a\sum _{i = \ell }^\infty i C_i - k \left( C_1^2 + \sum _{i=1}^{\infty } C_i C_1 \right) + b \sum _{i=2}^{\ell - 1}i C_i , \end{aligned}$$

31b $$\begin{aligned} \dot{C}_i&= k C_1 C_{i - 1} - k C_1 C_i - f(i) C_i, \quad i \ge 2, \end{aligned}$$

31c $$\begin{aligned} f(i)&= {\left\{ \begin{array}{ll} b, & i < \ell , \\ a, & i \ge \ell , \end{array}\right. } \end{aligned}$$

31d $$\begin{aligned} C_1(0)&=Q, \quad C_i(0) = 0, \quad \text {for} \quad i \ge 2. \end{aligned}$$

The total count of size 1 oligomers is conserved:

$$\begin{aligned} \sum _{i=1}^\infty i C_i = C_1(0) = Q. \end{aligned}$$

### Homogenizing the Atomization Model

We homogenize in an analogous way to the two previous models.

32a $$\begin{aligned} \dot{C}&= a \int _\ell ^\infty u \Theta (u,t) du - k C\left( C+ \int _1^\infty \Theta (u,t) du \right) + b \int _1 ^\ell u \Theta (u,t) du, \end{aligned}$$

32b $$\begin{aligned} \frac{\partial \Theta }{\partial t}&= -k C \frac{\partial \Theta }{\partial s} - f(s) \Theta (s,t) ,\quad s > 1, \end{aligned}$$

with conserved quantity

$$\begin{aligned} C + \int _1^\infty u \Theta (u,t) du = Q. \end{aligned}$$

Note that *Q* is finite which implies $\int _1^\infty u \Theta (u,t) du$ and $\int _1^\infty \Theta (u,t)du$ are bounded. This is biologically sound.

Note that System (32) no longer has a fission-free steady state. The only steady state is

$$\begin{aligned} Q&= C + k C^2 e^{\frac{-b}{kC}(\ell - 1)}\left( \frac{\ell }{a} - \frac{\ell }{b} + \frac{k C}{a^2} - \frac{k C}{b^2} \right) + \frac{k C^2}{b} \left( \frac{k C}{b} + 1 \right) , \\ \Theta (s)&= C e^{\frac{-b}{k C}(s - 1)}, \quad \quad \quad \quad s < \ell , \\ \Theta (s)&= Ce^{\frac{1}{kC}(b - b\ell + a\ell - as)}, \quad s \ge \ell . \end{aligned}$$

The total fission rate of this equilibrium is $\int _\ell ^\infty a \Theta (u,t)du =k C^2 e^{\frac{b}{kC}(1-\ell )}$, and gives us confidence that the atomization model has removed the fission-free, stalled wave equilibrium present in the previous models.

#### Discontinuities in the Fission Function and Initial Conditions

Similar to previous sections, we have a discontinuous fission function given in Equation (31c), and we choose the initial conditions to be Equation (8). Any solution with these initial conditions solves System (32) in the weak sense. We define $s^*$ analogously to Sect. (2.1.1) and define $t^*$ as the solution to $\int _0^{t^*} k C(\xi ) d\xi = \ell - 1$.

Due to the atomization term in Equation (32a), we need new variables: $n(t) = \int _{1}^\infty \Theta (u,t)du$ and $m(t) = \int _{1}^\infty u \Theta (u,t) du$. Assuming $s^*(t) < \ell$,

33a $$\begin{aligned} \dot{n}(t)&= k C^2 - bn, \end{aligned}$$

33b $$\begin{aligned} \dot{m}(t)&= kC (C + n) - bm. \end{aligned}$$

Next we show that a solution for $t^*$ can always be found.

### Stalled Wave is Eliminated in the Homogenized Atomization Model

For $s^*(t) < \ell$, System (32), Equations (33), conserved quantity C+m=Q, and initial conditions (8) create the new ODE system:

34a $$\begin{aligned} \dot{C}&= - k C^2 - k C n - b C + bQ, \end{aligned}$$

34b $$\begin{aligned} \dot{n}&= k C^2 - bn. \end{aligned}$$

We showed above that System (32) does not have a fission-free steady state. This gives us confidence we can always solve for $t^*$. To prove this, we begin by showing *C*(*t*) in System (34) is not near 0 for any *t*. If this is true, $\int _0^{\infty } k C(\xi )d\xi$ will grow without bound and a solution $t^*$ can be found for any ℓ.

#### Theorem 3

If b>0, k>0, and Q>0 then the flow of System (34) is within the trapping region defined by n=0, n=Q, $n = - C + \sqrt{\frac{bQ}{k}} + Q$, and $C = \hat{C}$, where $\hat{C}$ is the positive solution to $-k C^2 - k C Q - b C + bQ = 0$.

#### Proof

Consider the trapezoid in the *C*, *n*-plane defined by n=0, n=Q, $n = - C + \sqrt{\frac{bQ}{k}} + Q$, and $C = \hat{C}$.

We proceed by showing the flow of the system cannot leave the trapezoid through any of its edges. Along the n=0 edge, $\dot{n} = k C^2 > 0$. Along the n=Q edge of the trapezoid, $C < \sqrt{\frac{bQ}{k}}$, therefore $\dot{n} = k C^2 - b Q < 0$. Along the $C = \hat{C}$ edge, $\dot{C} = k \hat{C} (Q - n) > 0$ (because n<Q on that edge).

The dynamics along the sloped edge of the trapezoid always point inward. We show this by using a dot product with a vector normal to the edge: (-1,-1).

35 $$\begin{aligned} (\dot{C}, \dot{n}) \cdot (-1,-1)&= k C^2 + k C n + b C - b Q - k C^2 + bn\nonumber \\&= k C n + b C - b Q + b(-C + \sqrt{\frac{bQ}{k}} + Q)\nonumber \\&= k C n + b \sqrt{\frac{bQ}{k}} > 0. \end{aligned}$$

Note that $\hat{C} < Q$ and therefore our initial condition (*Q*, 0) is within the trapping region.

Therefore the flow of the system cannot leave the trapezoid and *C*(*t*) can never approach 0. □

With proof that a solution exists for $t^*$ in the homogenized atomization model, we finally consider the dynamics of System (32) for $t > t^*$. We define partial moments, recalling the derivative of Θ(s,t) may not exist at size ℓ and size $s^* > \ell$. $n_1(t) = \int _1^\ell \Theta (u,t) du$, $n_2(t) = \int _\ell ^{s^*} \Theta (u,t) du$, $m_1(t) = \int _1^\ell u \Theta (u,t) du$, and $m_2(t) = \int _\ell ^{s^*} u \Theta (u,t) du$. We note that taking the derivatives of these and using integration by parts results in the term $\Theta (\ell ,t)$ appearing. We denote $\Theta (\ell ,t) = \Theta _\ell$ for the remainder of the section. Using these partial moments in System (32) yields

36a $$\begin{aligned} \dot{C}&= a m_2 - k C (C + n_1 + n_2) + b m_1, \end{aligned}$$

36b $$\begin{aligned} \dot{n}_1&= -k C (\Theta _\ell - C) - b n_1, \end{aligned}$$

36c $$\begin{aligned} \dot{m}_1&= -k C (\ell \Theta _\ell - C - n_1) - b m_1, \end{aligned}$$

36d $$\begin{aligned} \dot{n}_2&= k C \Theta _\ell - a n_2, \end{aligned}$$

36e $$\begin{aligned} \dot{m}_2&= k C (\ell \Theta _\ell + n_2) - a m_2, \end{aligned}$$

36f $$\begin{aligned} C(0)&= Q, \quad n_1(0) = m_1(0) = n_2(0) = m_2(0) = 0, \end{aligned}$$

with conserved quantity $C + m_1 + m_2 = Q$.

### Delay Dynamics in the Homogenized Atomization Model

We evaluate $\Theta _\ell$ using Equations (32b), (31c) and the method of characteristics. For s<ℓ,

$$\begin{aligned} \frac{\partial \Theta }{\partial t} + k C \frac{\partial \Theta }{\partial s} = - b \Theta (s,t). \end{aligned}$$

**Fig. 7:**
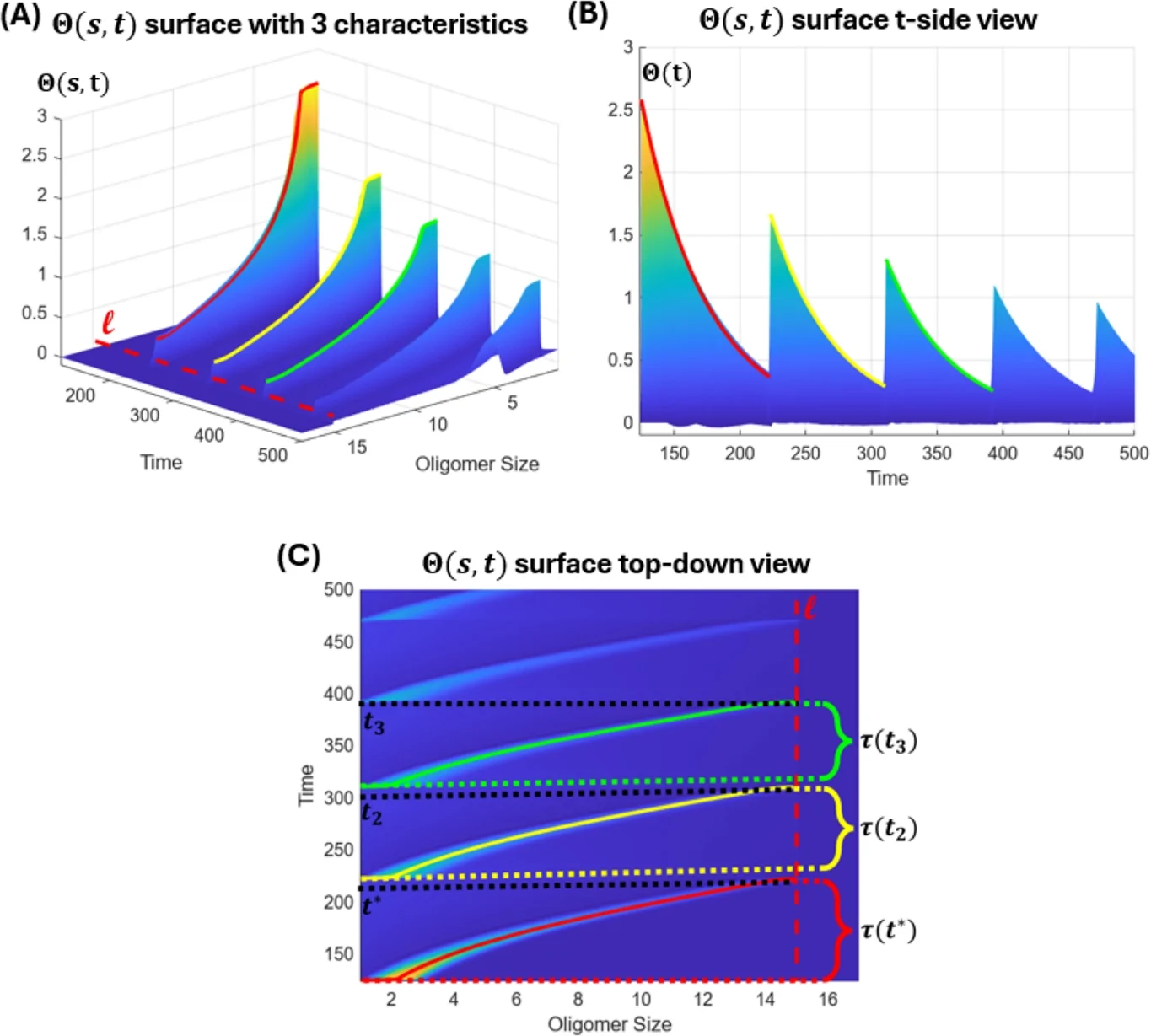
Numerical solutions for the homogenized atomization model. (A) Numerical simulation of the Θ(s,t) surface created by simulating *C*(*t*) using the sdDDE form of the atomization model and then using the resulting *C*(*t*) as a boundary condition for PDE Equation (32b). The sdDDE was solved using parameter set: ℓ=15, a=5, k=1, b=0.02, and Q=12. Three characteristics are graphed on top of the surface. Note the perspective has large time and large oligomer sizes in the foreground to avoid the largest peak obscuring the others. (B) The same surface from a side-on perspective. From this perspective, the exponential decay behavior in time along the characteristics is clear. (C) The same surface from a top-down perspective. At time $t_i$ one can see the corresponding characteristic intersects size ℓ=15. Following the characteristics back through size and time, the associated $\tau (t_i)$ is revealed as the time in the past where the characteristic intersects size 1

If $\frac{d s(t)}{dt} = k C(t)$, then $\frac{d \Theta }{dt} = -b \Theta (s(t),t)$. Therefore, $\Theta (s(t),t) = Ae^{-b t}$ for some *A* along characteristics. For $t < t^*$ any characteristic that intersects (ℓ,t) also intersects (*s*, 0) for s>0, where the initial condition is Θ(s,0)=0. Therefore $A = 0 = \Theta _\ell$ for $t < t^*$. For $t > t^*$, we can integrate along the characteristics to define a delay term τ(t).

$$\begin{aligned} \int _{t - \tau (t)}^t k C(\xi )d\xi = \ell - 1 \end{aligned}$$

Intuitively, this delay can be thought of as the time it takes a cohort of oligomers to grow from size 1 to size ℓ. When $t > t^*$, the characteristics will intersect both (ℓ,t) and (1,t-τ(t)) where $\Theta (1,t - \tau (t)) = C(t - \tau (t))$. Thus, $C(t - \tau (t)) = A e^{-b(t - \tau (t))}$ which implies $A = C(t - \tau (t))e^{b (t - \tau (t))}$ and $\Theta _\ell = C(t - \tau (t))e^{-b \tau (t)}$ for $t > t^*$. An example of these characteristic curves on a Θ(s,t) surface can be seen in Fig. 7.

Finally, we have shown the homogenized atomization model is equivalent to a state-dependent delay-differential Equation (sdDDE) of threshold-type, with threshold value ℓ-1:

37a $$\begin{aligned} \dot{C}&= a m_2 - k C (C + n_1 + n_2) + b m_1, \end{aligned}$$

37b $$\begin{aligned} \dot{n}_1&= -k C (C(t - \tau )e^{-b \tau } - C) - bn_1, \end{aligned}$$

37c $$\begin{aligned} \dot{m}_1&= - k C (\ell C(t - \tau )e^{-b \tau } - C - n_1) - bm_1, \end{aligned}$$

37d $$\begin{aligned} \dot{n}_2&= k C C(t - \tau )e^{-b \tau } - a n_2, \end{aligned}$$

37e $$\begin{aligned} \dot{m}_2&= k C (\ell C(t - \tau )e^{-b \tau } + n_2) - a m_2, \end{aligned}$$

37f $$\begin{aligned} \int _{t - \tau (t)}^t&k C(\xi ) d\xi = \ell -1, \end{aligned}$$

37g $$\begin{aligned} C(0)&= Q, \quad n_1(0) = m_1(0) = n_2(0) = m_2(0) = 0, \end{aligned}$$

with conserved quantity $C + m_1 + m_2 = Q$.

The existence of this sdDDE provides valuable insight into the process of mitochondrial fission. The solutions of the Leinheiser et al. model displayed oscillatory behavior, and they were numerically proved to be the result of a Hopf bifurcation. However, the reason a Drp1-dependent mitochondrial fission model undergoes a Hopf bifurcation is unclear from the ODE formulation in Leinheiser et al. The homogenized atomization model (which displays qualitatively similar behavior) contains delay terms which predispose the system to oscillatory behavior. Furthermore, the sdDDE formulation of Drp1-dependent mitochondrial fission highlights that the oscillations first observed in Leinheiser et al. are a consequence of the delay in oligomer construction on the outer mitochondrial membrane. Also, the sdDDE handles the discontinuous initial conditions without resorting to a weak formulation as we saw with the PDE version.

### The Hopf Bifurcation of the Homogenized Atomization Model

**Fig. 8:**
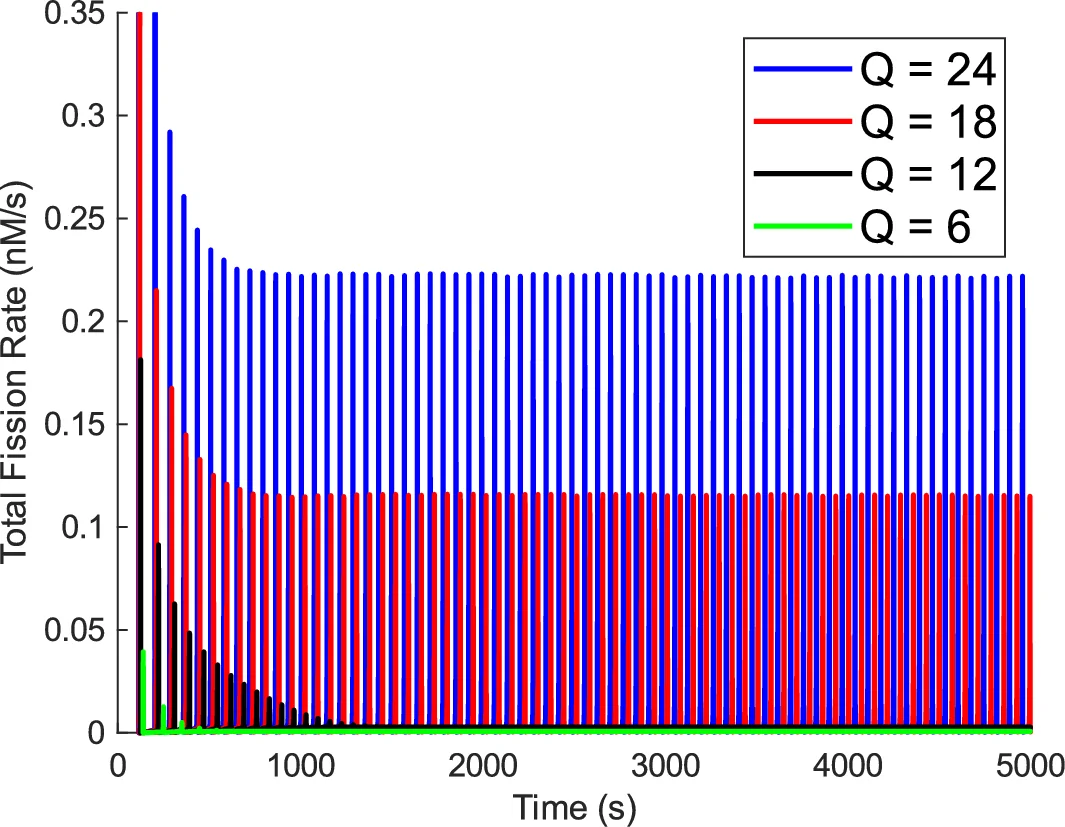
Numerical simulations (Shampine 2005) of the homogenized atomization model. The parameter values are a=5, k=1, b=0.02, and ℓ=15 with various values of *Q* as indicated. Note, these numerical solutions are qualitatively similar to Fig. 1

Numerical solutions for the homogenized atomization model (System 37) showcase that for different values of *Q*, i.e. the material pool, the total fission rate exhibits damped oscillations that reach a steady state or stable oscillations that appear to not reach a steady state (Fig. 8). Note that while the scale of these solutions is different from Fig. 1, the qualitative structure of these solutions is the same. The structure indicates the homogenized atomization model produces an analogous Hopf bifurcation. To confirm this, we compute the eigenvalues of the system. Note, the steady state equations of System (37) are transcendental because of the $e^{-b \tau }$ terms. We continue with linearization knowing the steady states will be calculated numerically. The conserved quantity is used to eliminate the equation for $\dot{m}_2$ and so that we have a non-hyperbolic system. Note that one need not consider the state-dependent properties of τ near equilibrium, following the theorems from Cooke and Huang (1996).

The full process of checking the bifurcation structure of chosen parameters is as follows. First, a parameter set is chosen. Then the steady state values of each variable are numerically calculated using Newton’s Method. Those steady state values are plugged into the characteristic equation and the eigenvalues are calculated. The characteristic equation is also transcendental and therefore must be numerically calculated. Due to the complexity of this process, it is difficult to determine the exact value when bifurcation occurs. We chose a standard set of parameters within which a single parameter was varied and a range between which the bifurcation occurs is identified (Fynaardt 2026).

**Fig. 9:**
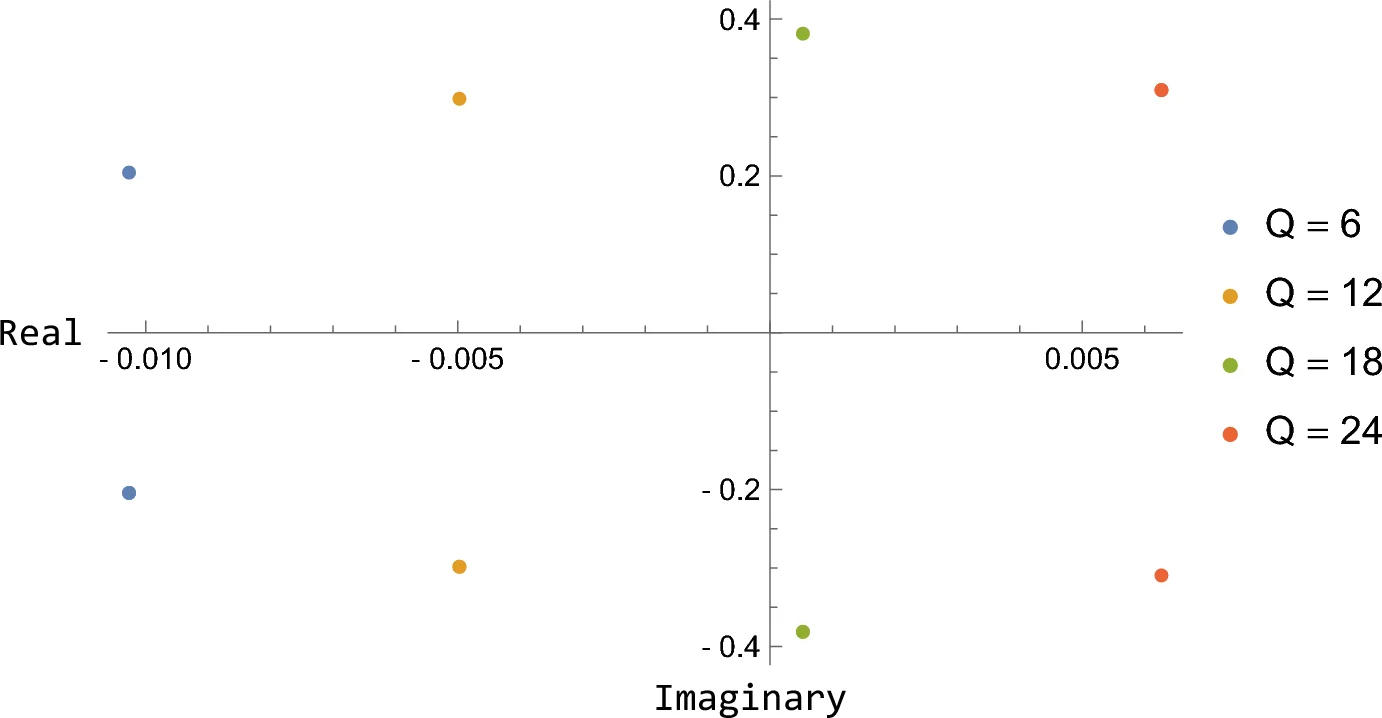
The eigenvalues with the largest real part of the characteristic equation of the homogenized atomization model with parameters a=5, k=1, b=0.02, and ℓ=15, with varying *Q*. The figure shows the pairs of imaginary eigenvalues passing over the imaginary axis as parameter *Q* passes from 12 to 18, showing a Hopf bifurcation occurs at that point. We note the change in the real part of the eigenvalues from Q=12 to Q=18, where the bifurcation is predicted to occur, is approximately 5 thousandths

**Fig. 10:**
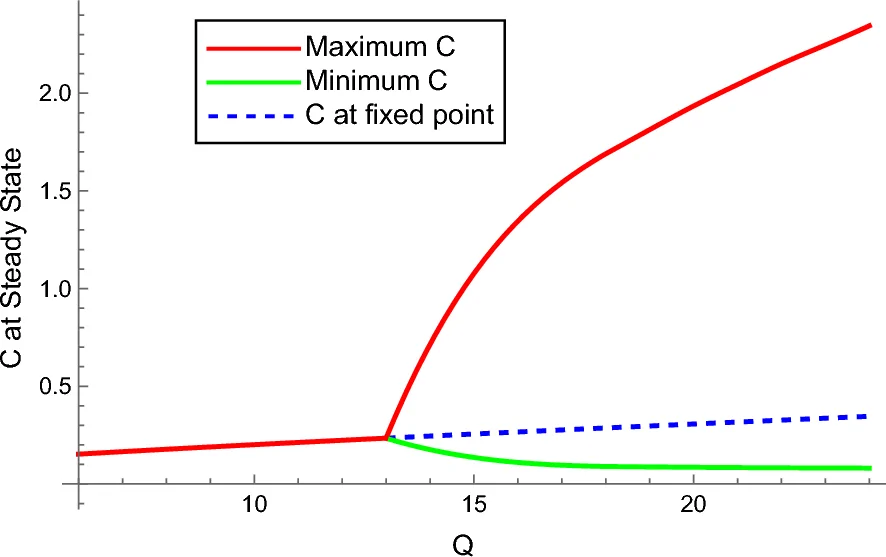
A bifurcation diagram of the atomization model showing the change in the steady state of C as Q is varied. The structure of a pitchfork bifurcation is clear. We note this diagram shows the bifurcation occurs near Q=13

Our parameter set was chosen to be similar to the original Leinheiser et al. model. As a result, a=5, k=1, b=0.02, and ℓ=15. For each calculation, these parameters were held constant while the parameter *Q* was varied over discrete values. The bifurcation is identified as the value for which the real part of the eigenvalue crosses the imaginary axis, following the canonical structure of a Hopf bifurcation (Fig. 9). The resulting bifurcation diagram appears in Fig. 10. In conclusion, the homogenized atomization model does indeed produce an analogous Hopf bifurcation to the Leinheiser et al. model. The biological implication is that the inherent delay behavior of oligomerization leads to oscillation in total fission rate.

## Discussion

Mitochondria are the primary producers of ATP and play a critical role in the regulation of cellular metabolism. Therefore, the maintenance of the mitochondria is crucial for the health of the cell. The mitochondrial population is maintained through the interplay of three main processes: mitophagy, fission, and fusion. In this study, we have chosen to focus on the regulation of fission. However, each process plays an important role, and future work should incorporate these elements. In response to metabolic or environmental stressors, mitochondria can enter a state of hyperfission which can impair ATP production and lead to programmed cell death. Therefore, studying the mechanisms and regulation of fission is critical to understand mitochondrial homeostasis.

In this work, we examine the mathematical model for Drp1-dependent mitochondrial fission from Leinheiser et al. (2024) and develop an sdDDE model for mitochondrial fission. Whilst developing the sdDDE, we generated a simplified model by removing the parameter $k_-$, disallowing the disassembly of oligomers on the mitochondrial membrane. The homogenization of this model leads to an advection PDE with non-local interactions via the boundary and velocity. One of the two model solutions is a stalled wave of middle sized oligomers which never reach a sufficient size to induce fission. With the initial conditions from Leinheiser et al., the solution for the simplified model is in the basin of attraction for this fission-free equilibrium. We show this stalled wave also exists in the original Leinheiser et al. model when $k_-$ is set to zero. This suggests that a method for dissassembly of the Drp1 oligomers is necessary to reduce the potentiality of a fission-free equilibrium. In all three models, the stability of each equilibrium is left as another future direction, as the theorems present in this work consider only specific initial conditions and not the basins of attraction for each equilibrium.

Since a mechanism which allows a stable fission-free equilibrium would be detrimental to mitochondrial homeostasis, we reincorporated $k_-$ into our mechanism with a bidirectional model. After homogenization, the bidirectional model also generates an advection PDE with non-local interactions via the boundary and velocity. Interestingly, even with the re-addition of $k_-$, the solutions for our homogenized bidirectional system persist in the basin of attraction of the stable, fission-free equilibrium. Note that in numerical simulations of the Leinheiser et al. model, $k_-$ greater than zero is enough to escape this basin of attraction. As part of the homogenization, we chose an approximation which omitted the diffusion term. We conjecture that an approximation that is an advection–diffusion model could destabilize the fission-free equilibrium consistent with the original Leinheiser et al. simulations. However, we chose to pursue a form of the PDE which preserves the delay dynamics since our focus is understanding the inherent delay-like behavior observed in the oscillatory numerical solutions.

We therefore propose an atomization model which includes an atomization term *b* instead of a dissociation term $k_-$. This atomization term allows oligomers to return to size one at any point in the building process. With this mechanism, we are able to derive an sdDDE of threshold type which eliminates the fission-free equilibrium.

Another possible strategy would be to explore other models for the building of Drp1 oligomers. The atomization model presented here requires oligomers to build one block at a time. This choice is consistent with the Leinheiser et al. model as well as the suggested mechanism described in Michalska et al. (2018) for Drp1. This Becker-Döring type mechanism has also been adopted in other models of cellular polymerization (for example Edelstein-Keshet and Ermentrout 1998). However, Strack et al. (2013) hypothesizes that larger size oligomers may also be able to combine on the mitochondrial surface. In that case, we would arrive at a Smoluchowski coagulation (or coagulation-fragmentation) model with fission-driven non-local feedback on the boundary condition. A straightforward calculation shows that such a model also has no fission-free equilibrium since middle size oligomers can continue to combine even when the pool of monomers is depleted.

The homogenized atomization model allows another look at the Hopf bifurcation observed in Leinheiser et al.  Analogous to that model, the total number of building blocks ($\mathcal {M}$ and *Q* respectively) is a bifurcation parameter. This qualitatively similar Hopf bifurcation recovered in the sdDDE corroborates that the oscillatory behavior of the Leinheiser et al. model is due to underlying delay dynamics. This suggests that mitochondrial fission may display functional oscillations due to the intrinsic delay in Drp1-oligomerization. We do not know of any studies which have directly measured the time course of fission rate under cellular stress with sufficient temporal resolution to observe such oscillations. Thus, their physiological relevance remains an important open question. While several quantities related to mitochondrial function have been observed to oscillate (for example, Aon et al. 2008; Porat-Shliom et al. 2014; Neufeld-Cohen et al. 2016 and Yu et al. (2021)), it is unclear whether the oscillations observed here could occur under physiological conditions. The models discussed in this paper suggest that the material pool for oligomers as well as the oligomerization kinetics are key parameters to explore experimentally in order to investigate oscillatory fission behavior.

Beyond the application to mitochondrial fission, the derivation of this sdDDE model provides insight into homogenization techniques. A homogenized PDE model with nonlocal information on the boundary condition and the velocity term is difficult to analyze. Further, a homogenization with diffusion disallows the use of the method of characteristics. Thus, the introduction of the atomization parameter served to circumvent the issues with diffusion terms while retaining the qualitative behavior of the original discrete system. Further analysis of sdDDEs of threshold type as approximations of comparable ODE systems could reveal the differences between systems with a dissociation term and systems with an atomization term.

These results provide further evidence that the timing and regulation of oligomer assembly are central determinants of mitochondrial dynamics. Since excessive or unregulated mitochondrial fission is implicated in cardiovascular disease, metabolic dysfunction, neurodegeneration, and cancer, understanding the temporal mechanisms governing oligomerization provides insight into how mitochondrial populations transition between healthy and pathological states. The atomization framework introduced here highlights the importance of regulatory mechanisms that continually reset or redistribute oligomer populations.

More broadly, this study demonstrates how homogenization techniques and sdDDE reductions can uncover latent delay structure in large mechanistic systems. The resulting models provide analytically tractable descriptions of nonlocal transport processes while preserving key biological dynamics that may otherwise be obscured in high-dimensional ODE systems. Beyond mitochondrial fission dynamics, the framework developed here may prove useful for understanding other intracellular assembly processes in which threshold formation times and delayed feedback play a fundamental role.

## Supplementary information

The code used to create figures and simulations have been included as supplementary files.

## Data Availability

This study did not generate or analyze any publicly archived datasets.

## Code Availability

All mathematical derivations and simulation methods are included as supplementary files.
